# Assessment of Clinical Time Intervals Along the Patient Journey with Overall Survival in Localized Osteosarcoma and Ewing Sarcoma: A 20-Year Tertiary Sarcoma Centre Experience

**DOI:** 10.3390/cancers18182927

**Published:** 2026-09-09

**Authors:** Roberta Laranga, Cristina Ferrari, Federica Zuccheri, Valentina Clementi, Giuseppe Bianchi

**Affiliations:** 1 Department of Orthopaedic Oncology, IRCCS Istituto Ortopedico Rizzoli, 40136 Bologna, Italy; federica.zuccheri@ior.it (F.Z.); valentina.clementi@ior.it (V.C.); giuseppe.bianchi@ior.it (G.B.); 2 Laboratory of Oncology Research and Functional Genomics, IRCCS Istituto Ortopedico Rizzoli, 40136 Bologna, Italy; 3 Department of Biomedical and Neuromotor Sciences (DIBINEM), University of Bologna, 40138 Bologna, Italy

**Keywords:** osteosarcoma, Ewing’s sarcoma, time to treatment initiation, clinical time intervals, overall survival, bone sarcoma, tertiary referral centre

## Abstract

Background: The impact of healthcare-related time intervals on outcomes in bone sarcomas remains controversial. We evaluated the impact of clinically defined post-referral, in-hospital care intervals on overall survival (OS) and their variation over two decades, in patients treated at a tertiary sarcoma centre. Methods: We retrospectively analysed 506 patients with localized osteosarcoma (*n* = 319) or Ewing’s sarcoma (*n* = 187) treated between 2002 and 2021. We defined three in-hospital time intervals: time to diagnosis (TToD; first specialist evaluation to diagnostic biopsy), time to treatment (TToT; biopsy to treatment initiation), and total post-referral patient journey time (PJT; first specialist evaluation to treatment). Multivariable Cox models adjusted for age, sex, tumour location, and surgical factors evaluated associations with OS. Results: Median PJT was longer in Ewing’s sarcoma than in osteosarcoma (17 vs. 14 days; *p* < 0.001), mainly because of a longer TToT. Over time, median PJT increased from 12 to 19 days (*p* < 0.001), driven by longer TToT, while TToD remained stable. Neither PJT nor TToD was associated with OS. Although longer TToT was associated with lower mortality risk, this was not confirmed after stratification by diagnostic period. Worse OS was independently associated with amputation, upper-extremity tumours, and intralesional margins. Conclusions: Within the specialized tertiary centre setting, longer in-hospital care timelines were not associated with poorer overall survival despite increasing over time, a finding that may reflect the growing complexity of multidisciplinary management exclusively during the post-referral phase.

## Simple Summary

Osteosarcoma and Ewing’s sarcoma are rare, aggressive bone cancers that mainly affect children, adolescents, and young adults. Early diagnosis and prompt treatment are crucial to the outcomes and survival of these patients. However, the effect of the time between diagnosis and treatment on patient survival is still unclear. In this study, we examined three specific post-referral, in-hospital time intervals during the diagnosis and treatment of patients with localized osteosarcoma and Ewing’s sarcoma who were treated at a specialized sarcoma centre. By using objectively recorded clinical milestones instead of patient-reported symptom onset, we aimed to offer a more precise assessment of care timelines. We then investigated whether these intervals were linked to overall survival, while also taking established clinical prognostic factors into account. Our findings add to the ongoing discussion about the importance of treatment timing in bone sarcoma care and offer insights into how to better organize specialized multidisciplinary referral pathways for patients with these rare cancers.

## 1. Introduction

Osteosarcoma and Ewing’s sarcoma are two rare and highly aggressive bone tumours that mainly affect children, adolescents, and young adults [1,2,3]. Over the past decades, significant advances in early detection and multimodal cancer therapies have led to improved overall survival rates in the general patient population. Nevertheless, prognosis remains poor for patients suffering from these musculoskeletal malignancies [4,5,6,7,8]. Among established prognostic factors, metastatic status at diagnosis, tumour biology, and response to treatment are consistently recognized as the strongest determinants of outcomes [9,10]. Despite the introduction of modern systemic therapies, the overall 5-year survival rate remains approximately 60–70% [6,11,12,13,14,15]. Because these tumours often present with nonspecific symptoms and are infrequently encountered in routine clinical practice, delays in diagnosis and referral to specialist centres remain a recognized challenge. Poor disease recognition among patients and healthcare providers further contributes to delays along the diagnostic and treatment pathways [16].

The time between diagnosis and initiation of treatment is one of the variables that may impact the outcome of patients. Several studies have investigated the association between diagnosis date, treatment initiation, and survival rates, achieving conflicting results. Some studies indicate a connection between longer intervals and inferior outcomes, while others have failed to demonstrate a significant relationship between treatment timing and survival [12,16,17,18]. Interpreting these results is challenging due to high heterogeneity among existing studies, which often feature disparate patient samples, the inclusion of metastatic cases, and inconsistent definitions of time intervals [19]. Therefore, determining the specific prognostic significance of individual stages within the diagnostic and treatment pathways remains difficult [20].

The importance of prompt referral to specialized sarcoma centres is well-documented as a decisive strategy for providing high-quality management through accurate diagnosis, thorough multidisciplinary assessment, correct staging, and coordinated treatment planning [21,22]. However, even following referral, there is still a chance for variations in clinical timelines, even in the dedicated sarcoma facilities, and the prognostic implications of the identified discrepancies are still poorly understood.

To address these limitations, this study evaluated three objectively defined clinical time intervals in a cohort of patients with localized osteosarcoma and Ewing’s sarcoma treated at a single tertiary referral centre over a 20-year period. In contrast to previous studies relying on patient-reported symptom onset, our analysis was based on objectively recorded clinical milestones along the specialist care pathway, including the first specialistic evaluation, the histopathological diagnosis, and treatment commencement. In addition, we restricted our analysis to patients with localized disease in order to minimize the confounding effect of metastatic status at diagnosis.

The primary aim of this study was to evaluate the association between clinically defined time intervals along the diagnostic and treatment pathway and overall survival in patients with localized osteosarcoma and Ewing’s sarcoma. The secondary objectives were: (1) to characterize the distribution of these clinical time intervals in the overall cohort and according to histological subtype and (2) to assess temporal trends in these intervals over the study period.

## 2. Materials and Methods

### 2.1. Study Design and Population

This is a retrospective cohort study comprising a total of 506 patients (319 with osteosarcoma and 187 with Ewing’s sarcoma) who were diagnosed and treated for primary and localized sarcoma at the Rizzoli Orthopaedic Institute (IOR), a single tertiary sarcoma referral centre, between 2002 and 2021. Histological diagnosis was performed according to the WHO 2020 classification [23], by experienced IOR pathologists. Patients were treated using a multimodal approach according to the European guidelines [24]. Follow-up data and patient status were updated through 30 September 2025, using a digital archive. The study was approved by the Local Ethical Committee (CE-AVEC: 436/2025/Oss/IOR) on 24 July 2025.

### 2.2. Definition of Clinical Time Intervals

Clinical data were extracted from institutional medical records. Three clinically relevant milestones were defined: first specialist evaluation (T1), histological diagnosis following biopsy (T2), and initiation of oncological treatment (T3).

**Figure 1:**
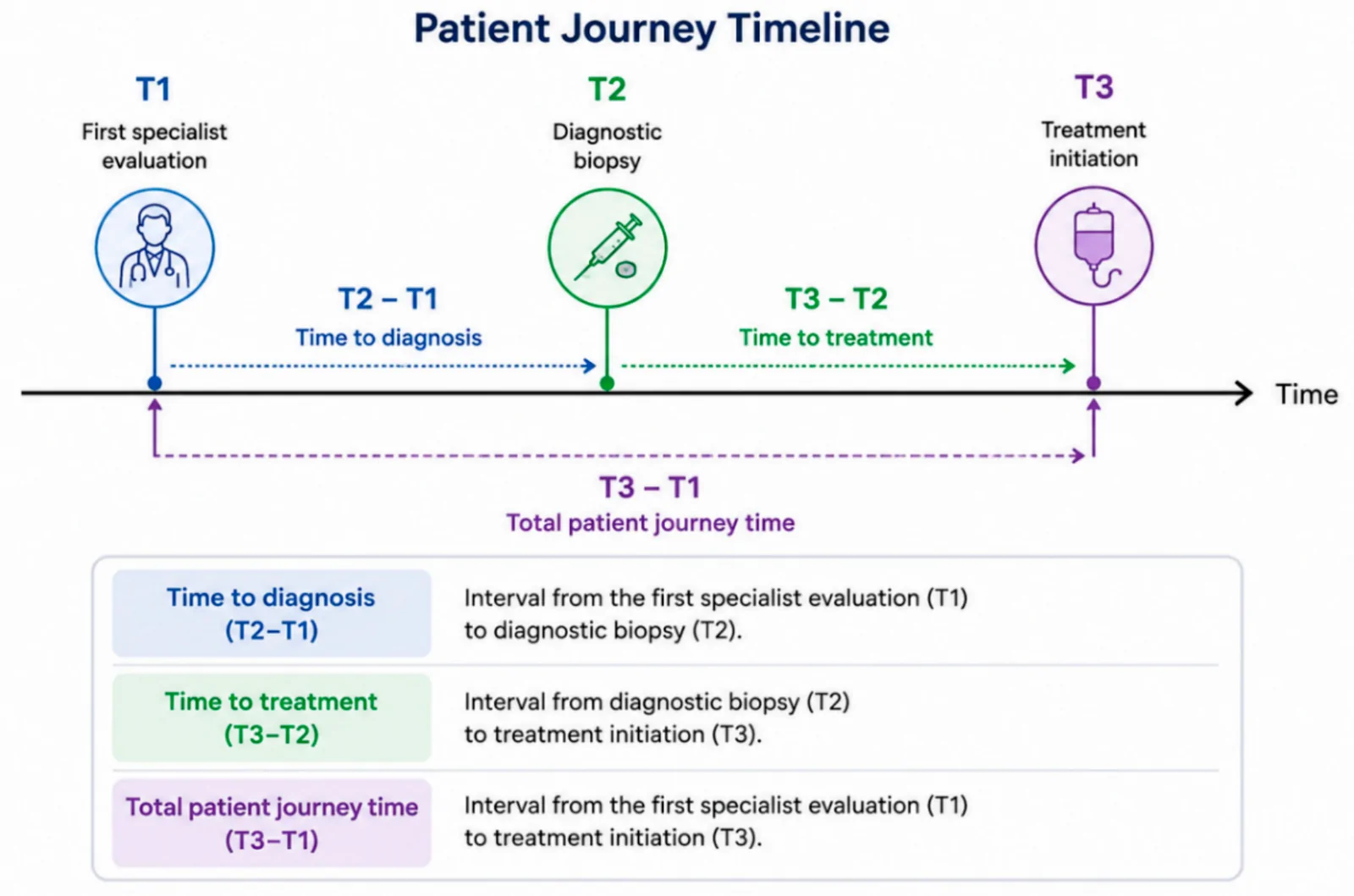
Patient journey timeline: total patient journey time (PJT = T3 − T1), time to diagnosis (TToD = T2 − T1) and time to treatment (TToT = T3 − T2).

Based on these timepoints, three clinical intervals were calculated: time from first evaluation to diagnosis (time to diagnosis, TToD = T2 − T1), time from diagnosis to treatment initiation with definitive surgery or chemotherapy (time to treatment, TToT = T3 − T2), and total patient journey time (PJT = T3 − T1) (Figure 1). All intervals were derived from documented clinical events rather than from patient-reported symptom onset (Figure 1).

### 2.3. Clinical Variables

Baseline variables included age, sex, histological subtype (osteosarcoma vs. Ewing’s sarcoma), tumour location (upper extremity, axial skeleton, pelvis and lower extremity), biopsy procedure (needle biopsy, incisional biopsy), type of surgery (limb-salvage, amputation, or other intralesional surgery), and surgical margin status (wide, radical, marginal and intralesional).

### 2.4. Statistical Analysis

Continuous variables were summarized as medians and interquartile ranges (IQRs), while categorical variables were reported as frequencies and percentages. The Mann–Whitney *U* test, Kruskal–Wallis test and chi-square test were used as appropriate. For continuous variables compared between two groups using the Mann–Whitney U test, effect sizes were calculated as the rank-biserial correlation (r) to quantify the magnitude of the differences.

Overall survival (OS) was defined as the time from histological diagnosis (T2) to death from any cause or last follow-up. Survival estimates were calculated using the Kaplan–Meier method, and survival differences between groups were assessed using the log-rank test. The association between time intervals and OS was evaluated using Cox proportional hazard regression models. To simultaneously adjust for potential confounders, all pre-specified covariates were included in a multivariable Cox proportional hazard regression model. Two complementary modelling strategies were applied. First, each time interval was analysed separately in univariable and multivariable Cox regression models. Second, diagnostic and treatment intervals were included simultaneously in multivariable models to assess their independent association with OS while accounting for potential overlap between time intervals. The same full-model adjustment strategy was applied across all analyses. To account for potential immortal time bias associated with the time from histological diagnosis to treatment initiation (T2 − T3), we verified that no deaths occurred within this specific interval in our cohort.

Histological subtype was first included as a covariate in the multivariable Cox models and was subsequently explored through stratified analyses according to tumour subtype.

All multivariable models were adjusted for age, sex, histological subtype, tumour location, surgical margins, and type of surgery. Hazard ratios (HRs) with 95% confidence intervals (CIs) are reported and graphically displayed using forest plots. A two-sided *p*-value < 0.05 was considered statistically significant.

Temporal changes and distribution patterns are graphically represented with Boxplots and LOWESS-smoothed curves. All analyses were performed using StataSE 18 (StataCorp LLC, College Station, TX, USA).

## 3. Results

### 3.1. Patient Characteristics and Clinical Features

A total of 506 patients diagnosed between 2002 and 2021 were included in the study. The cohort was divided into two subgroups according to their clinical diagnosis: osteosarcoma or Ewing’s sarcoma. The baseline demographic and clinical characteristics of the osteosarcoma patients (N = 319) and the Ewing’s sarcoma cohort (N = 187) are summarized in Table 1 and Table 2, respectively.

### 3.2. Clinical Time Intervals

Clinical time intervals according to histological subtype and in the overall cohort are reported in Table 3.

Patients with Ewing’s sarcoma experienced significantly longer TToT intervals than patients with osteosarcoma (median: 13 vs. 8 days, *p* < 0.001, effect size r = 0.245). Consequently, PJT was also significantly longer in Ewing’s sarcoma (median: 17 vs. 14 days, *p* < 0.001, effect size r = 0.251). In contrast, TToD did not differ significantly between histological subtypes (median: 5 days in both groups, *p* = 0.320, effect size r = 0.044).

### 3.3. Overall Survival

OS rates at 60 and 120 months were 72.8% and 67.8% for Ewing’s sarcoma and 73.5% and 67.8% for osteosarcoma, respectively (Supplementary Table S1, Supplementary Figure S1). OS rates did not differ significantly between patients with Ewing’s sarcoma and osteosarcoma (log-rank *p* = 0.686).

**Figure 2:**
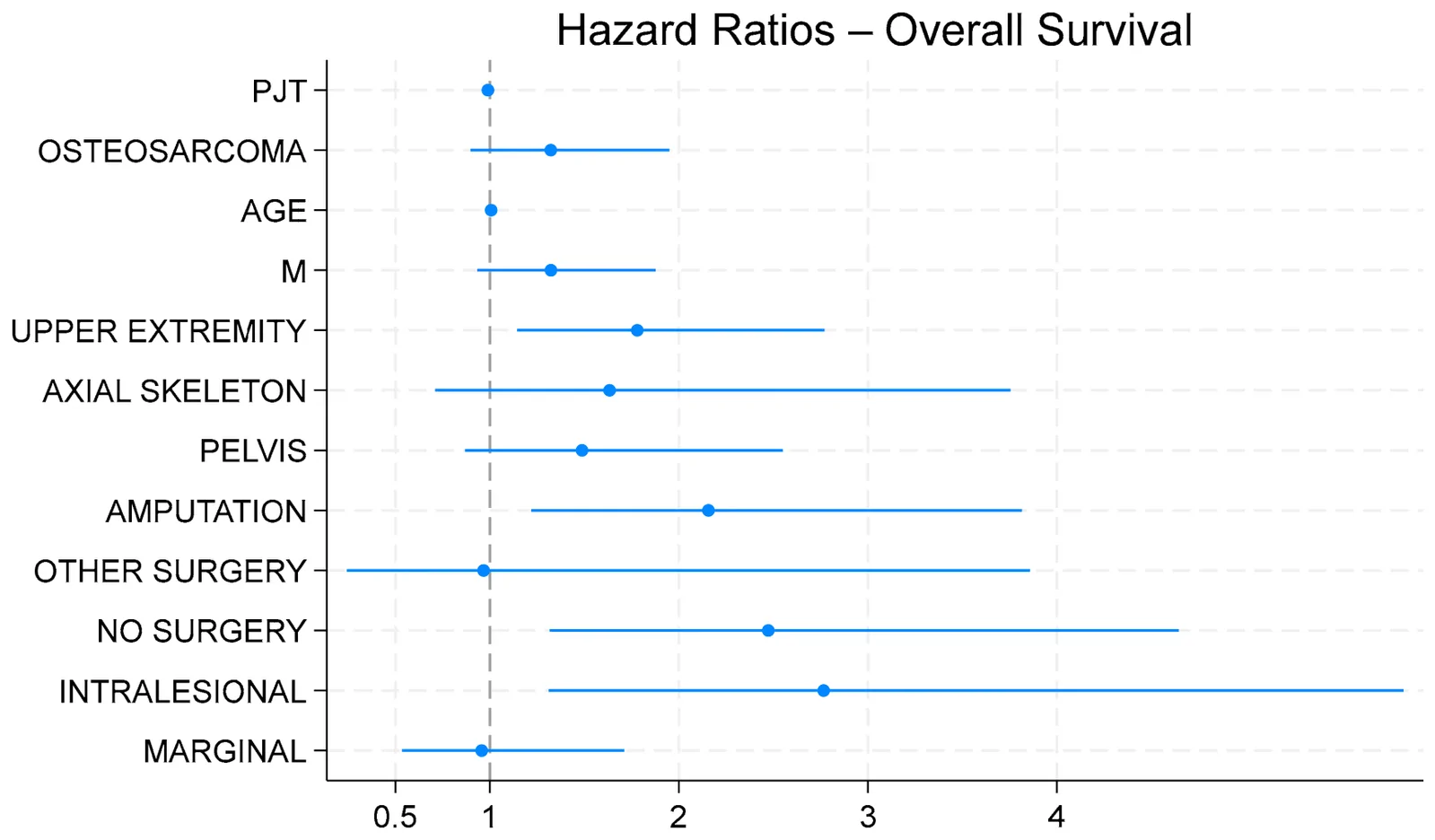
Multivariable Cox regression forest plot for total patient journey time (PJT). The vertical dashed line indicates the line of null effect (Odds Ratio = 1.0). Horizontal bars represent the 95% confidence intervals.

Multivariate analyses revealed that PTJ was not significantly associated with mortality (HR 0.99, 95% CI 0.97–1.01, *p* = 0.094) (Table 4, Figure 2).

Concerning tumour site, the upper-extremity location was significantly associated with poorer survival than the lower extremity (HR 1.77, 95% CI 1.14–2.76, *p* = 0.011).

Regarding treatment factors, surgical amputation (HR 2.16, 95% CI 1.21–3.81, *p* = 0.008), the absence of surgery (HR 2.47, 95% CI 1.31–4.64, *p* = 0.005) and intralesional margins (HR 2.76, 95% CI 1.31–5.83, *p* = 0.008) were independently associated with an increased risk of death. No significant associations were observed for sex, histological subtypes, age, marginal margins, or other tumour locations.

**Figure 3:**
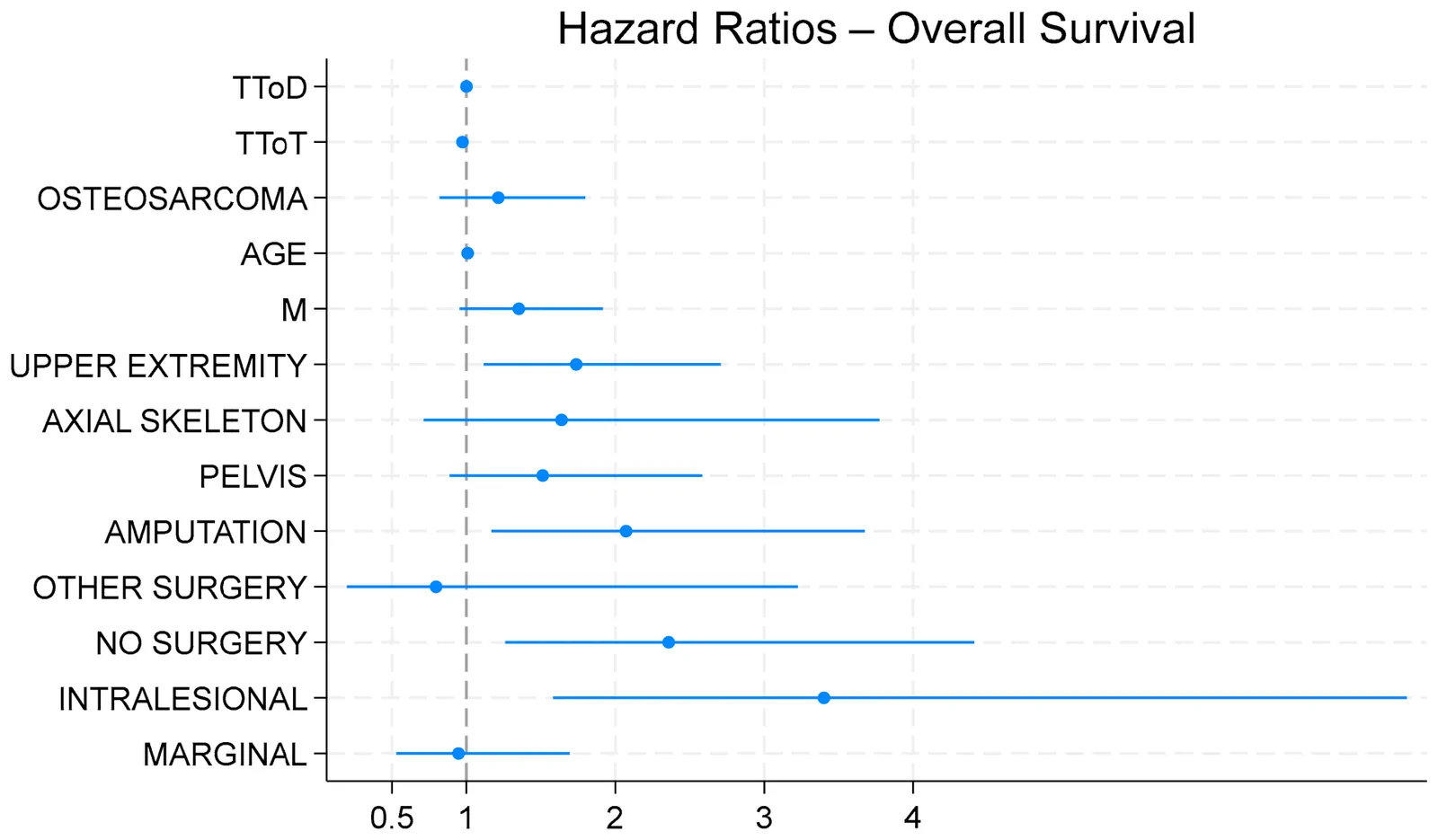
Multivariable Cox regression forest plot for time to diagnosis (TToD) and time to treatment (TToT). The vertical dashed line indicates the line of null effect (Odds Ratio = 1.0). Horizontal bars represent the 95% confidence intervals.

To avoid masking potential differential effects of specific care phases, TToD and TToT times were entered separately into a second multivariable model (Table 5, Figure 3).

TToD was not associated with OS (HR 1.00, 95% CI 0.99–1.01, *p* = 0.980). Conversely, a longer TToT was associated with a modest reduction in mortality risk (HR 0.97, 95% CI 0.95–0.99, *p* = 0.009). Tumour location in the upper extremity was significantly associated with OS (HR 1.73, 95% CI 1.11–2.70; *p* = 0.015). Surgical amputation (HR 2.07, 95% CI 1.16–3.67, *p* = 0.013), the absence of surgery (HR 2.35, 95% CI 1.26–4.41, *p* = 0.007), and intralesional margins (HR 3.40, 95% CI 1.58–7.31, *p* = 0.002) were also associated with an increased risk of death. No significant associations were observed for diagnosis, age, sex, tumour location in the pelvis or axial skeleton, or marginal margins.

For each of the two clinical entities, we analysed the impact of time intervals on survival in separate stratified multivariable analyses (Supplementary Tables S1 and S2).

In the localized osteosarcoma cohort (N = 319), no intervals demonstrated a significant association with OS (Supplementary Table S2). Conversely, in the Ewing’s sarcoma cohort (N = 187), while total journey time and time to diagnosis remained non-significant, TToT was significantly associated with a modest reduction in mortality risk (HR 0.96, 95% CI 0.93–0.99; *p* = 0.016, Supplementary Table S3).

### 3.4. Temporal Trends in Clinical Time Intervals

For temporal trend analyses, we divided the cohort into three diagnostic periods: 2002–2007 (*n* = 200, 39.4%), 2008–2014 (*n* = 178, 35.0%), and 2015–2021 (*n* = 130, 25.6%).

The median PJT increased over time, from 12 days (IQR 9–15) in 2002–2007 to 17.5 days (IQR 13–28) in 2008–2014, and 19 days (IQR 15–25) in 2015–2021. The median TToD remained relatively stable across periods, ranging from 4 to 5 days. Conversely, median TToT increased from 6 days (IQR 4–10) to 13 days (IQR 9–19) (Table 6).

**Figure 4:**
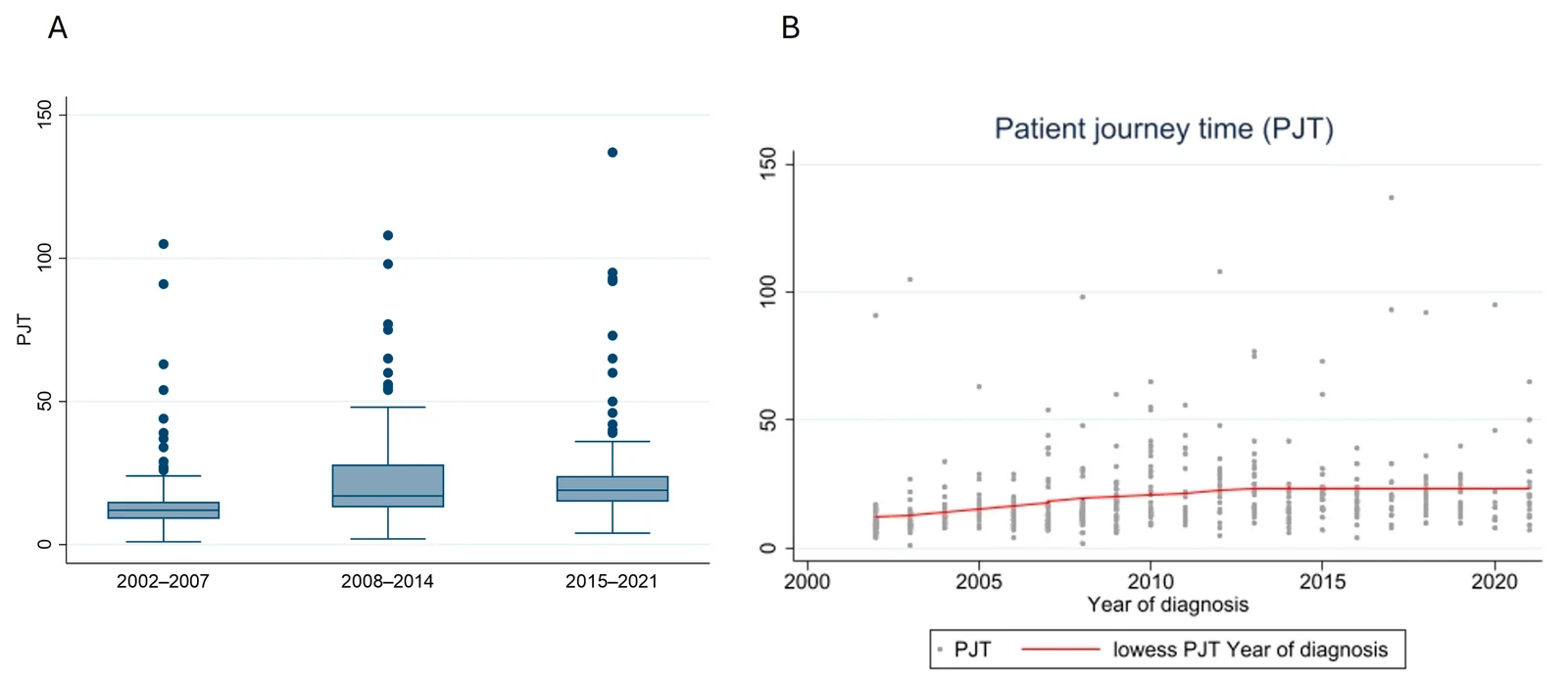
Temporal trends in patient journey time (PJT). (**A**) Boxplots showing the distribution of time to diagnosis across diagnostic periods. (**B**) LOWESS-smoothed curve illustrating temporal changes over the study period.

**Figure 5:**
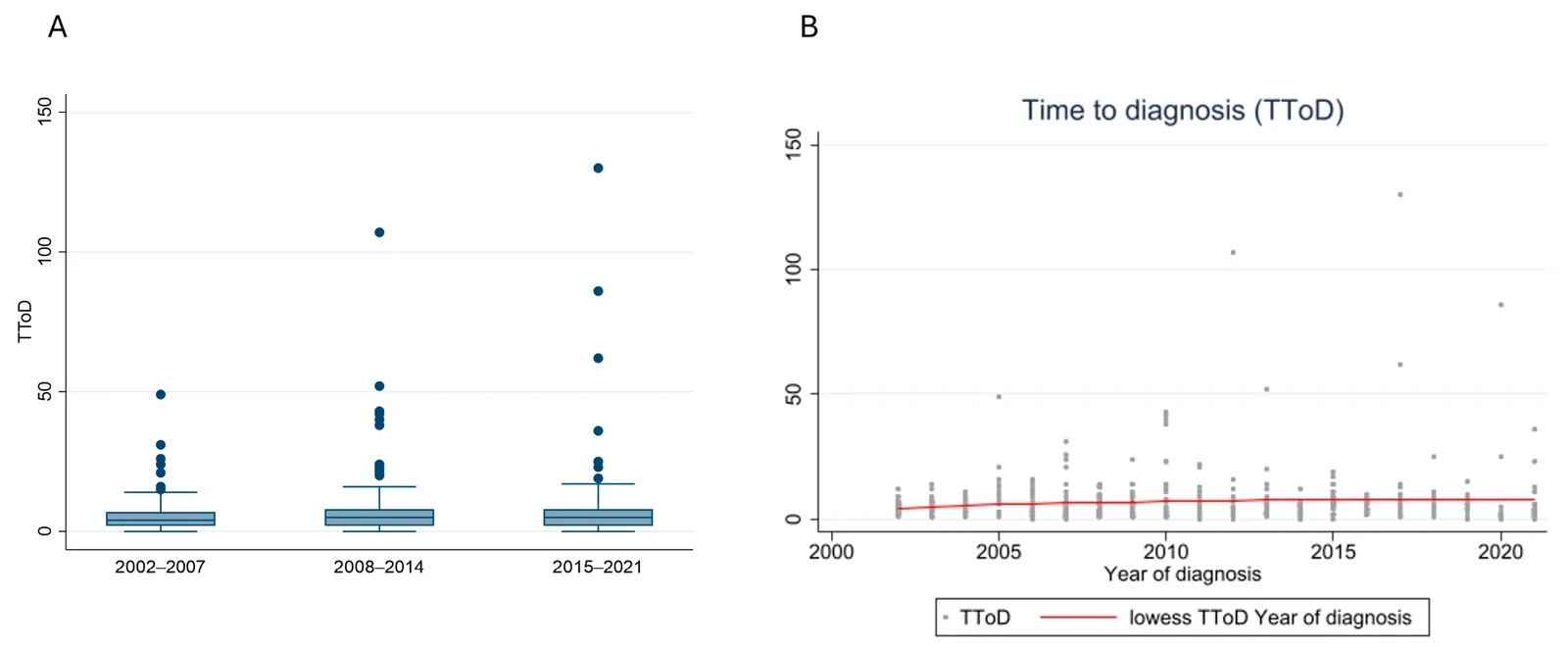
(**A**) Boxplots of clinical time intervals by time to diagnosis (TToD); (**B**) LOWESS temporal trends of clinical time intervals of time to diagnosis (TToD).

**Figure 6:**
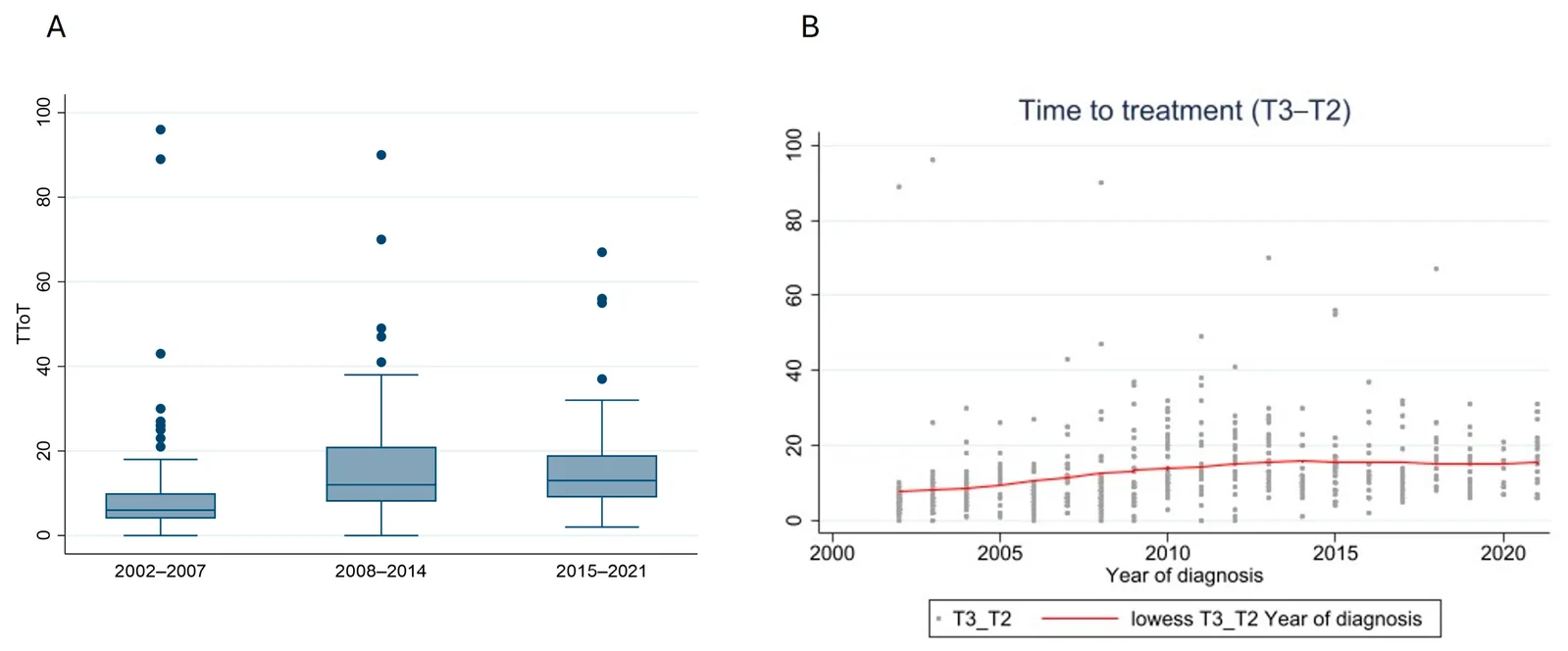
(**A**) Boxplots of clinical time intervals by time to treatment (TToT); (**B**) LOWESS temporal trends of clinical time intervals of time to treatment (TToT).

We found a significant difference across diagnostic periods for PJT (χ^2^ = 90.9, *p* < 0.001) and TToT (χ^2^ = 102.0, *p* < 0.001). In contrast, no significant differences were observed for TToD (χ^2^ = 1.1, *p* = 0.59). Boxplots and LOWESS-smoothed trend curves confirmed these findings (Figure 4, Figure 5 and Figure 6).

### 3.5. Association Between Clinical Time Intervals and Overall Survival

**Figure 7:**
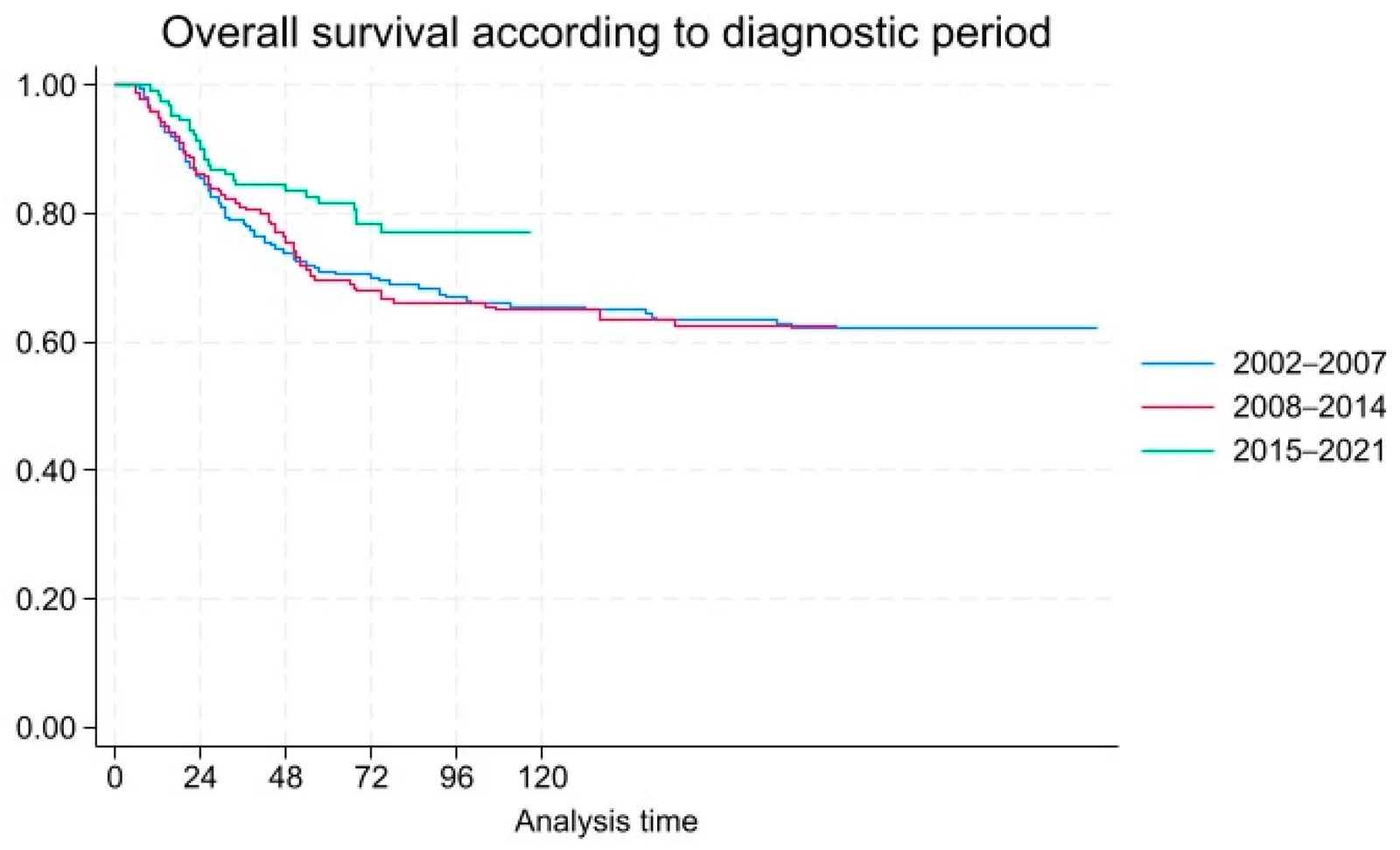
Overall survival estimation according to diagnostic period (log-rank *p* = 0.0930).

Kaplan–Meier estimates showed no significant differences in OS across diagnostic periods (log-rank *p* = 0.0930) (Figure 7).

No significant association was observed between TToD, TToT, or PJT and OS in any of the three periods analysed (Table 7).

## 4. Discussion

This study evaluated clinical intervals within the specialist care pathway of patients with localized osteosarcoma and Ewing’s sarcoma treated at a tertiary sarcoma centre over two decades. As these metrics exclusively capture in-hospital, post-referral operational milestones rather than the entire pre-hospital patient journey, three principal findings emerged. First, the overall pathway from first specialist evaluation to treatment initiation was short, with a median patient journey time (PJT) of 15 days, although patients with Ewing’s sarcoma had a longer PJT than those with osteosarcoma, principally because of a longer time to treatment (TToT). Second, PJT and TToT increased significantly across diagnostic periods, whereas time to diagnosis (TToD) remained stable. Third, neither PJT nor TToD was associated with overall survival after adjustment for demographic, anatomical, and surgical factors. Although longer TToT was associated with a small reduction in mortality in the pooled analysis, this association was not reproduced in period-specific models.

The absence of a clear association between the measured intervals and OS is consistent with part of the existing bone sarcoma literature, in which the relationship between diagnostic delay and outcome has been heterogeneous. In a prospective multicentre study of children and adolescents with Ewing’s tumours, Brasme et al. reported that a longer time to diagnosis was not associated with metastatic status or survival [17]. More-recent reviews underline that studies on diagnostics and treatment management in bone sarcoma differ substantially in their definitions, starting points, populations, and endpoints, and that statistically robust survival effects are not consistently demonstrated [19]. These discrepancies are important because a patient-reported symptom interval, a primary-care interval, a referral interval, and a post-referral specialist interval represent biologically and organizationally distinct phases. The present study specifically evaluated the latter phase and therefore complements, rather than replaces, research addressing delays before presentation or referral.

This distinction may also explain why the present results differ from evidence in more-common malignancies. A large systematic review and meta-analysis found that treatment delays were associated with higher mortality across several common cancers and treatment modalities [20]. However, those estimates cannot be directly transferred to localized bone sarcomas, which are rare, biologically heterogeneous, and managed through multimodal protocols. In addition, the intervals observed in this cohort were generally measured in days rather than in weeks or months. The findings therefore suggest that modest variation within a relatively rapid specialist pathway may have less prognostic importance than tumour biology, extent of disease, treatment response, and the feasibility of adequate local control. Nevertheless, our results should be interpreted with caution, especially in the context of prolonged delays and metastatic disease.

The unexpected link between longer treatment times and lower mortality in the pooled model and the Ewing’s sarcoma subgroup requires careful interpretation. The effect size was small, was not observed for total PJT, and disappeared when analyses were stratified by diagnostic period. A plausible explanation is residual confounding or a “waiting-time paradox”: patients with more-aggressive clinical features, greater symptom burden, or an urgent need for treatment may have been prioritized for earlier therapy, whereas clinically stable patients may have undergone more-extensive staging and planning [25]. Moreover, although both OS and TToT were measured from the date of histological diagnosis, TToT represents a post-baseline variable and should therefore be interpreted as an association rather than a causal determinant of survival. Accordingly, the observed hazard ratio should be regarded as hypothesis-generating rather than causal evidence [26]. Furthermore, we specifically verified that no patient events (deaths) occurred between the time of histological diagnosis and the initiation of treatment, confirming that immortal time bias did not influence the evaluation of the treatment interval in our cohort.

The progressive increase in TToT, from a median of 6 days in 2002–2007 to 13 days in 2015–2021, was the principal driver of the increase in PJT. The stability of TToD suggests that access to biopsy and diagnostic procedures within the centre remained efficient, while the post-biopsy phase became longer. The reasons for this change were not captured in the available data. It may reflect the increasing complexity of contemporary sarcoma care, including multidisciplinary review, completion of staging, centralized pathological assessment, treatment protocol selection, pre-treatment organ function assessment, fertility-preservation counselling, and coordination among medical oncology, surgery, radiotherapy, and supportive services. These activities are integral to specialist management recommended by sarcoma guidelines [21,24], but their contribution to the observed temporal trend should be confirmed directly rather than assumed. Importantly, longer post-referral inpatient care timelines were not associated with poorer overall survival despite increasing over time.

The histology-specific differences are clinically plausible. Patients with Ewing’s sarcoma had a longer TToT than those with osteosarcoma, despite a similar TToD. Ewing’s sarcoma frequently requires coordination of systemic treatment, molecular confirmation, staging, and planning of subsequent local therapy within an integrated protocol, all of which may contribute to longer pre-treatment intervals [2,14,27,28]. Nevertheless, with the present data, we are not able to identify which components of the pathway are involved in the additional days. Future analyses linking interval data to specific workflow steps would clarify these differences.

By contrast, several anatomical and surgical variables were associated with OS. Upper-extremity tumour location, amputation, and intralesional margins were independently associated with higher mortality in the multivariable models. Furthermore, while the complete absence of surgery is strongly associated with an increased risk of mortality, it is an exceptionally rare event in this localized cohort, reflecting the strict selection and treatment standards of our tertiary centre. These findings underscore the primary prognostic importance of disease characteristics and local treatment factors over short fluctuations in specialist care timelines. However, they also require cautious interpretation. Amputation is unlikely to be intrinsically responsible for poorer survival; rather, it likely identifies patients with larger, anatomically complex tumours involving neurovascular structures or those for whom limb-salvage surgery was not feasible [9,16]. Similarly, intralesional margins may reflect challenging tumour anatomy and are directly relevant to local disease control [29]. Established osteosarcoma series have consistently shown that tumour-related factors, response to chemotherapy, and adequacy of local control are major determinants of outcome [9,16]. Although axial and pelvic locations typically carry the worst prognosis, our findings unexpectedly highlight an independent impact associated with upper-extremity tumours, diverging from the conventional literature. This likely stems from unique anatomical hurdles: the complex relationships between the tumour and adjacent neurovascular, tendinous, and muscular structures frequently limit the ability to achieve clear margins without compromising limb function [30,31]. These technical constraints, alongside unmeasured variables like tumour size and deep compartmental location, merit cautious interpretation and validation in independent cohorts.

This study has several strengths. It includes a relatively large series for two rare malignancies, was conducted in a high-volume referral centre, and spans 20 years of clinical practice. Restriction to localized disease reduced the confounding effect of metastatic presentation. The use of documented milestones avoided recall bias associated with patient-reported symptom onset and provided reproducible interval definitions. The analysis also evaluated the total pathway and its component phases separately, adjusted for relevant clinical factors, compared histological subtypes, and examined whether associations were consistent across calendar periods. This approach aligns with the Aarhus statement, which emphasizes precisely defined timepoints and transparent reporting in cancer pathway research [22].

Several limitations must also be acknowledged. First, the retrospective, single-centre design limits causal inference and external generalizability, particularly to institutions lacking dedicated sarcoma pathways. Second, in response to the retrospective, long-term nature of our cohort, crucial prognostic parameters for bone sarcomas—specifically exact tumour size and the histological response to neoadjuvant chemotherapy (such as Huvos grading)—were not uniformly available across all historical records and could not be incorporated into the multivariable models. Similarly, detailed data regarding specific treatment regimens, radiotherapy, comorbidities, and socioeconomic factors were unavailable. Consequently, residual confounding is likely. Furthermore, the study did not measure symptom onset, first healthcare contact, referral time, or reasons for delay and therefore cannot address the complete pre-hospital patient journey. The small numbers in some anatomical and surgical categories reduced precision, while patients diagnosed in the most recent period had shorter potential follow-ups.

Clinically, these findings suggest that, within the relatively short post-referral treatment intervals achieved at a specialized sarcoma centre, modest variations in TToT were not associated with poorer OS.

In conclusion, while our 20-year single-centre cohort provides a robust real-world evaluation of in-hospital clinical time intervals in bone sarcomas, these findings must be interpreted with strict regard to their post-referral context. Given the retrospective design and the multiple potential unmeasured confounders inherent to long-term observational datasets, our results should not be viewed as diminishing the general importance of timely diagnosis or pre-hospital referral. Rather, they highlight that, within an established specialist framework, disease biology and local-treatment factors play the dominant prognostic role. Future prospective multicentre studies incorporating standardized pre-hospital and in-hospital interval definitions, detailed clinical and biological data, and time-dependent analyses are needed to distinguish harmful pre-referral delays from appropriate time invested in specialistic diagnostic accuracy and multidisciplinary treatment planning.

## 5. Conclusions

Based on our 20-year tertiary sarcoma centre experience, the conclusions of this study are summarized below:

- Timeline Trends: Post-referral clinical timelines in the management of localized osteosarcoma and Ewing’s sarcoma have progressively lengthened over the past two decades, driven primarily by longer intervals between histological diagnosis and treatment initiation.
- Prognostic Impact: Despite these timeline shifts, variations in these in-hospital care intervals were not associated with worse overall survival.
- Relative Importance: Within a specialized sarcoma referral centre, post-referral operational timeline variations have limited prognostic relevance compared with established biological and surgical risk factors.
- Clinical Implications: These results support the safety and effectiveness of contemporary in-hospital multidisciplinary care pathways, suggesting that necessary organizational complexity can be managed without compromising oncological outcomes during the specialist phase of care.

## Figures and Tables

**Table 1:** Osteosarcoma baseline demographic and clinical characteristics of the cohort (N = 319).

Variable	Category	N	%
Sex	Female	121	38.0
	Male	198	62.0
Age (years)	16 (12–23)		
Tumour location	Upper extremity	31	9.72
	Axial skeleton	5	1.57
	Pelvis	14	4.39
	Lower extremity	269	84.33
Biopsy procedure	Needle biopsy	188	59.0
	Incisional biopsy	95	29.0
	Unknown	36	12.0
Surgical procedure	Resection	291	91.2
	Amputation	21	6.6
	Other	2	0.6
	No surgery	5	1.6
Surgical margins	Wide	252	79
	Radical	21	6.6
	Marginal	33	10.4
	Intralesional	8	2.5
	NA (no surgery)	5	1.5

Data are presented as *n* (%) unless otherwise specified. Age is reported as median (interquartile range, IQR). Not available (NA).

**Table 2:** Ewing’s sarcoma baseline demographic and clinical characteristics of the cohort (N = 187).

Variable	Category	N	%
Sex	Female	54	28.8
	Male	133	71.2
Age (years)	16 (8–24)		
Tumour location	Upper extremity	32	17.1
	Axial Skeleton	18	9.6
	Pelvis	47	25.1
	Lower extremity	90	48.1
Biopsy procedure	Needle biopsy	109	58.0
	Incisional biopsy	48	26.0
	Unknown	30	16.0
Surgical procedure	Resection	141	75.4
	Amputation	4	2.14
	Other	5	2.67
	No surgery	37	19.79
Surgical margins	Wide	123	66
	Radical	4	2.2
	Marginal	13	7
	Intralesional	10	5
	NA (no surgery)	37	19.8

Data are presented as *n* (%) unless otherwise specified. Age is reported as median (interquartile range, IQR). Not available (NA).

**Table 3:** Clinical time intervals according to histological subtype and in the overall cohort.

Variable	Osteosarcoma (N = 319)	Ewing’s Sarcoma (N = 187)	Overall Cohort (N = 506)
TToD	5 (2–7)	5 (2–8)	5 (2–7)
TToT	8 (6–13)	13 (7–20)	10 (6–16)
PJT	14 (10–20)	17 (10–26)	15 (11–22)

Abbreviations: TToD, time to diagnosis; TToT, time to treatment; PJT, total patient journey time. Data are presented as median (interquartile range [IQR]).

**Table 4:** Multivariable Cox regression analysis of overall survival (N = 506).

Variable	Hazard Ratio	95% Confidence Interval	*p*-Value
PJT, per day	0.99	0.97–1.01	0.094
Diagnosis			
Ewing’s sarcoma	Reference	—	—
Osteosarcoma	1.32	0.89–1.94	0.159
Age (per year)	1.01	0.99–1.02	0.296
Sex			
Female	Reference	—	—
Male	1.32	0.93–1.87	0.117
Tumour Location			
Lower extremity	Reference	—	—
Upper extremity	1.77	1.14–2.76	**0.011**
Axial skeleton	1.63	0.71–3.75	0.248
Pelvis	1.48	0.86–2.55	0.149
Surgical Procedure			
Resection	Reference	—	—
Amputation	2.16	1.21–3.81	**0.008**
Other	0.96	0.24–3.85	0.962
None	2.47	1.31–4.64	**0.005**
Surgical Margins			
Wide margins	Reference	—	—
Intralesional margins	2.76	1.31–5.83	**0.008**
Marginal margins	0.96	0.53–1.71	0.880
Radical margins ^1^	Not estimable	—	—
None	Not estimable	—	—

^1^ Note: Radical margins could not be estimated due to the absence of death events in this specific subgroup. Bold values indicate statistical significance (*p* < 0.05).

**Table 5:** Multivariable Cox regression analysis of overall survival (diagnostic and treatment times entered separately) (N = 506).

Variable	Hazard Ratio	95% Confidence Interval	*p*-Value
TToD	1.00	0.99–1.01	0.980
TToT	0.97	0.95–0.99	**0.009**
Diagnosis			
Ewing’s sarcoma	Reference	—	—
Osteosarcoma	1.21	0.82–1.79	0.332
Age (per year)	1.01	0.99–1.02	0.162
Sex			
Female	Reference	—	—
Male	1.35	0.95–1.91	0.091
Tumour Location			
Lower extremity	Reference	—	—
Upper extremity	1.73	1.11–2.70	**0.015**
Axial skeleton	1.64	0.71–3.77	0.246
Pelvis	1.51	0.88–2.58	0.129
Surgical Procedure			
Resection	Reference	—	—
Amputation	2.07	1.16–3.67	**0.013**
Other	0.79	0.19–3.22	0.749
None	2.35	1.26–4.41	**0.007**
Surgical Margins			
Wide margins	Reference	—	—
Intralesional margins	3.40	1.58–7.31	**0.002**
Marginal margins	0.95	0.53–1.69	0.855
Radical margins ^1^	Not estimable	—	—
None	Not estimable	—	—

^1^ Note: Radical margins could not be estimated due to the absence of death events in this specific subgroup. Bold values indicate statistical significance (*p* < 0.05). Abbreviations: TToD, time to diagnosis; TToT, time to treatment.

**Table 6:** Temporal trends in clinical time intervals according to diagnostic period (N = 506).

Variable	2002–2007	2008–2014	2015–2021	*p*-Value *
TToD	4 (2–7)	5 (2–8)	5 (2–8)	0.59
TToT	6 (4–10)	12 (8–21)	13 (9–19)	<0.001
PJT	12 (9–15)	17.5 (13–28)	19 (15–25)	<0.001

* The *p*-values are from Kruskal–Wallis tests. Abbreviations: TToD, time to diagnosis; TToT, time to treatment; PJT, total patient journey time.

**Table 7:** Multivariable Cox regression models of temporal trends in clinical time intervals according to diagnostic period (N = 506).

Diagnostic Period	TToD	*p*-Value	TToT	*p*-Value	PJT	*p*-Value
2002–2007	1.01 (0.97–1.06)	0.380	0.98 (0.94–1.02)	0.300	0.99 (0.96–1.02)	0.608
2008–2014	1.00 (0.97–1.02)	0.804	0.97 (0.94–1.01)	0.121	0.99 (0.96–1.01)	0.293
2015–2021	1.00 (0.98–1.02)	0.877	0.99 (0.94–1.05)	0.901	1.00 (0.98–1.02)	0.926

Cox proportional hazards models were adjusted for diagnosis, age, sex, tumour location, surgical procedure, and surgical margins. Values are expressed as hazard ratios with confidence intervals. Abbreviations: TToD, time to diagnosis; TToT, time to treatment; PJT, total patient journey time.

## Data Availability

The data that support the findings of this study are not publicly available due to their containing information that could compromise the privacy of research participants but are available from the corresponding author (R.L.) upon reasonable request.

## Supplementary Materials

The following supporting information can be downloaded at https://www.mdpi.com/article/10.3390/cancers18182927/s1, Figure S1: Overall survival estimation by histology; Table S1. Overall survival estimation stratified by diagnosis; Table S2: Multivariable Cox regression analysis of overall survival in localized Ewing Sarcoma (*n* = 187); Table S3: Multivariable Cox regression analysis of overall survival in localized Osteosarcoma (*n* = 319).
