# Supplementary material for: Assessment of Clinical Time Intervals Along the Patient Journey with Overall Survival in Localized Osteosarcoma and Ewing Sarcoma: A 20-Year Tertiary Sarcoma Centre Experience
Source: Cancers (Basel). 2026 Sep 9;18(18):2927. doi: 10.3390/cancers18182927 (PMC13604304; doi:10.3390/cancers18182927)
Supplement: Supplementary file 1 [file cancers-18-02927-s001.zip › cancers-4502480-supplementary.pdf]

Table S1. Overall survival estimation stratified by diagnosis.

Time (months)	Ewing sarcoma (diagnosis 1)	Osteosarcoma (diagnosis 2)
12	94.7% (95% CI 90.3–97.1)	96.6% (93.9–98.1)
24	84.6% (78.6–89.0)	88.4% (84.3–91.4)
60	72.8% (65.8–78.6)	73.5% (68.2–78)
120	67.8% (60.5–74.1)	67.8% (62.2–72.8)

LOG RANK TEST Pr>chi2 = 0.6864

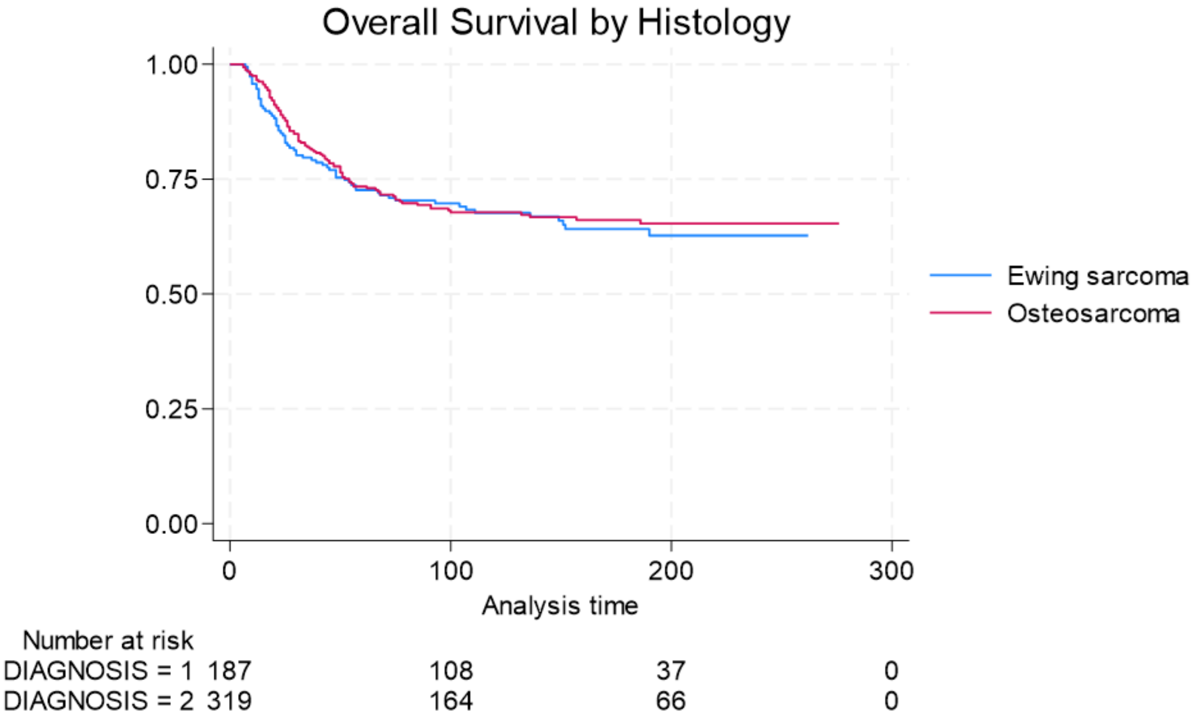

Figure S1: Overall survival estimation by histology.

Table S2. Multivariable Cox regression analysis of overall survival in localized Ewing Sarcoma (n=187).

**Variable**	**Hazard Ratio (HR)**	**95% Confidence Interval (CI)**	**p-value**
**Model 1:**			
PJT, *per day*	0.98	0.96 – 1.01	0.212
**Model 2:**			
TToD, *per day*	1.02	0.99 – 1.05	0.193
TToT, *per day*	0.96	0.93 – 0.99	**0.016**
**Demographics & Clinical Factors**[1]			
Age (*per year*)	1.01	0.99 – 1.04	0.169
Sex			
Female	*Reference*	—	—
Male	1.00	0.56 – 1.78	0.994
Tumor Location			
Lower extremity	*Reference*	—	—
Upper extremity	1.85	0.93 – 3.70	0.082
Axial skeleton	1.05	0.36 – 3.08	0.933
Pelvis	1.14	0.53 – 2.41	0.740
Surgical Procedure			
Resection	*Reference*	—	—
Amputation	1.31	0.174 – 9.95	0.789
Others	0.70	0.111 – 4.43	0.707
No surgery	2.24	1.07– 4.69	**0.033**
Surgical Margins			
Wide margins	*Reference*	—	—
Intralesional margins	3.55	1.14 – 11.04	**0.028**
Marginal margins	0.79	0.24 – 2.50	0.659
Radical margins[2]	Not estimable	—	–
No surgergical margins	Not estimable	—	–

Note: [1] HRs and *p*-values for clinical factors are extracted from Model 2; estimates from Model 1 were virtually identical. [2] Radical margins and no surgical margins could not be estimated due to the absence of events in this subgroup. Bold values indicate statistical significance ($p < 0.05$). *Abbreviations:* TToD, time to diagnosis; TToT, time to treatment; PJT, total patient journey time.

Table S3. Multivariable Cox regression analysis of overall survival in localized Osteosarcoma (n=319).

**Variable**	**Hazard Ratio (HR)**	**95% Confidence Interval (CI)**	**p-value**
**Model 1:**			
PJT, *per day*	0.99	0.97 – 1.01	0.355
**Model 2:**			
TToD, *per day*	0.99	0.98 – 1.01	0.714
TToT, *per day*	0.98	0.95 – 1.01	0.226
**Demographics & Clinical Factors**[1]			
Age (*per year*)	1.01	0.99 – 1.02	0.463
Sex			
Female	*Reference*	—	—
Male	1.70	0.61 – 2.73	**0.027**
Tumor Location			
Lower extremity	*Reference*	—	—
Upper extremity	1.43	1.77 – 2.66	0.254
Axial skeleton	4.58	1.06 – 19.75	**0.041**
Pelvis	2.22	1.03 – 4.78	**0.042**
Surgical Procedure			
Resection	*Reference*	—	—
Amputation	2.23	1.20 – 4.13	**0.010**
Others	4.82	0.63 – 36.5	0.128
No surgery	3.60	0.93 – 13.84	0.062
Surgical Margins			
Wide margins	*Reference*	—	—
Intralesional margins	2.53	0.72 – 8.83	0.145
Marginal margins	1.00	0.51 – 1.95	0.999
Radical margins	Not estimabile	–	–
No surgical margins	Not estimabile	–	–

Note: [1] HRs and *p*-values for clinical factors are extracted from Model 2; estimates from Model 1 were virtually identical. [2] Radical margins and no surgical margins could not be estimated due to the absence of events in this subgroup. Bold values indicate statistical significance ($p < 0.05$). *Abbreviations:* TToD, time to diagnosis; TToT, time to treatment; PJT, total patient journey time.
